# Academic health sciences libraries' outreach and engagement with North American Indigenous communities: a scoping review

**DOI:** 10.5195/jmla.2023.1616

**Published:** 2023-07-10

**Authors:** Allison Cruise, Alexis Ellsworth-Kopkowski, A. Nydia Villezcas, Jonathan Eldredge, Melissa L. Rethlefsen

**Affiliations:** 1 allison91b@gmail.com, Health Sciences Library & Informatics Center, University of New Mexico, Albuquerque, NM.; 2 AEllsworthKopkowski@salud.unm.edu, Health Sciences Library & Informatics Center, University of New Mexico, Albuquerque, NM.; 3 AVillezcas@salud.unm.edu, College of Population Health, University of New Mexico, Albuquerque, NM.; 4 JEldredge@salud.unm.edu, Health Sciences Library & Informatics Center, University of New Mexico, Albuquerque, NM.; 5 mlrethlefsen@gmail.com, Health Sciences Library & Informatics Center, University of New Mexico, Albuquerque, NM.

**Keywords:** Scoping review, Indigenous populations, outreach, community engagement, public participation, academic health sciences libraries

## Abstract

**Objective:**

We sought to identify trends and themes in how academic health sciences libraries in the United States, Canada, and Mexico have supported engagement and outreach with Native Americans, Alaska Natives, First Nations, and Indigenous peoples, in or from those same countries. We also sought to learn and share effective practices for libraries engaging with these communities.

**Methods:**

We conducted a scoping review utilizing Arksey and O'Malley's framework for scoping reviews and followed principles from JBI Manual for Evidence Synthesis. We searched seven bibliographic databases, E-LIS (Eprints in Library and Information Science repository), and multiple sources of grey literature. Results were screened using Covidence and Google Sheets. We reported our review according to the PRISMA and PRISMA-S guidelines. We determined types of interventions used by academic health sciences libraries in engagement with our included populations, the level of public participation reached by these interventions, what partnerships were established, and what practices emerged.

**Results:**

Database searching returned 2,020 unique results. Additional searching resulted in 211 further unique results. Full text screening of relevant articles found 65 reports meeting criteria for inclusion. Data extraction was conducted on these programs to identify partners, intervention type, and evaluation method. The programs were categorized using the IAP2 Spectrum of Public Participation.

**Conclusion:**

Our scoping review found that many programs were health information trainings and did not move beyond informing the public with little further involvement. The need for sustained funding, greater community participation and more publishing on engagement and outreach are discussed.

## INTRODUCTION

Systemic and structural racism has pervaded European-American society's interactions with Native American communities since the early 1500s. US Federal policies have institutionalized these forms of racism, and libraries have been embedded in these dominant European-American patterns of racism [1, 2]. While it is important to recognize these structures, academic health sciences libraries must move beyond an empty recognition of systemic issues which institutions and information resources have so often been built upon [3, 4]. Engaging in reckoning or anti-racist work and research in academic health sciences libraries is a continued commitment that should extend beyond improving library spaces to library research and outreach as well [5]. Academic library engagement that improves health literacy and health outcomes with underserved communities through this type of meaningful engagement is one way in which the oft-homogenized field of health sciences librarianship can subvert systemic issues and systems of oppression [6, 7].

Native Americans, Alaska Natives, First Nations, and Indigenous peoples face significant health challenges and disparities, while also possessing unique assets, strengths, and resilience [8]. Colonialism, systemic racism, and governments not upholding treaties or not meeting their trust relationship obligations have all contributed to the health disparity gap that persists [9]. Lower levels of health literacy and decreased access to health information have been shown to contribute to higher rates of hospitalizations, less participation in health screenings, and higher rates of emergency room utilization [10]. Strategizing ways to reach, support, and mutually engage with their communities has been a vital part of health sciences librarians' efforts to promote health equity. Moving away from health disparity research that centers solely on lifestyle choices and behavior changes towards engagement, while recognizing the historical and current inequities provides one way in which health science and information professionals can contribute to improving the health outcomes of these special populations [11].

A number of academic health sciences librarians have attempted to provide information services for Native communities, particularly through the efforts of the U.S. National Library of Medicine (NLM) and the National Network of Libraries of Medicine (NNLM; now the Network of the National Library of Medicine) [12]. There is an increased and continued need, however, for academic health sciences libraries to reach underserved communities to work towards providing needed health information and improving health literacy. Furthermore, academic health science libraries that are located in areas either near, or on Native lands can help to reduce health disparities through sustained collaboration, outreach, and engagement efforts with tribal nations. Improving health literacy through such efforts helps academic health sciences libraries improve their reach while serving their community partners and larger populations. These efforts also support the goal of Healthy People 2030 [13].

We conducted a scoping review to identify trends and themes in how academic health sciences libraries have supported community engagement and outreach with Native American, Alaska Native, First Nations, and Indigenous communities. Of particular interest were efforts that produced sustainable programs and relationships as such efforts align more closely with a Community Based Participatory Research (CBPR) methods approach or what has been defined as culturally safe research with Native Americans, Alaska Natives, First Nations, and Indigenous peoples [14].

Through this scoping review, we also sought to identify potential gaps in academic health sciences libraries' outreach and engagement to Native American, Alaska Native, First Nations, and Indigenous populations, and to share findings of effective, culturally appropriate practice.

## METHODS

This scoping review's methodology was guided by Arksey and O'Malley's framework for scoping reviews and conducted using the principles from the JBI Manual for Evidence Synthesis [15, 16]. PRISMA 2020 and PRISMA-S were followed to ensure complete reporting for the overall review and the search, respectively [17–19]. The review protocol was registered in OSF prior to beginning the search [20]. Adaptations to the protocol are described below.

We sought to answer the following research questions:

- Intervention types: What types of interventions are used by North American academic health sciences libraries when engaging with Native American, Alaska Native, First Nations, and Indigenous communities?
- Public participation: What is the level of public participation reached in these outreach/engagement interventions?
- Partnerships: What partnerships are established?
- Effective practices: What effective practices emerge from these interventions?

### Inclusion and Exclusion Criteria

We sought to identify reports that met the following inclusion criteria:

- Participants: Native American, Alaska Native, First Nations, and Indigenous students, community members, staff, and/or faculty were the primary participants. In this context, we considered these populations to include all peoples indigenous to lands currently occupied by Canada, Mexico, and the United States, whether or not residing on reservations.
- Library Participants: The identified community engagement effort was performed by an academic health sciences library in the US, Canada, or Mexico.
- Intervention(s): A description and/or evaluation of community engagement efforts was present, including, but not limited to, outreach programs, pipeline and other training programs, local collaborations with large-scale government projects, or the development of websites or information resources designed for communities.

We sought to include all types of information sources, both published and unpublished, in all languages. For languages other than English, Spanish, and Portuguese, we translated titles, abstracts, and full text using Google Translate with additional translation when required.

We excluded community engagement or outreach programs led by general academic, public, hospital, association, or government libraries. We also excluded reports describing community engagement or outreach programs which had an academic health sciences librarian as an additional participant, but where the project was not led by an academic health sciences library. In addition, we excluded all internal library training and all programs outside of the geographic scope of the review, such as reports in which either the Indigenous populations or the academic health sciences libraries were in countries other than Canada, Mexico, and the United States.

### Search Strategy

To identify reports that met our criteria, we searched the following seven bibliographic databases: Ovid MEDLINE ALL, Academic Search Complete (EBSCOhost), ERIC (ProQuest), Education Research Complete (EBSCOhost), LISA: Library and Information Science Abstracts (ProQuest), LISTA: Library, Information Science, and Technology Abstracts (EBSCOhost), and LILACS (https://lilacs.bvsalud.org/). We also searched a library and information science repository, E-LIS (http://eprints.rclis.org/). The searches were executed on February 14, 2022. We developed the Ovid MEDLINE search first, using Campbell's filter for studies related to Indigenous peoples of the United States as a base search [21], to which we added additional English and Spanish-language terms related to Indigenous peoples from Canada, Mexico, and the United States (see https://osf.io/6wt5a for an example of identified terms). These terms were combined with terms related to academic health sciences libraries, including some derived from Giustini et al. [22]. Complete bibliographic database search strategies are available in searchRxiv [23–30].

We sought unpublished and other grey literature material by searching and browsing in a combination of specific journals, conference proceedings, and websites. We identified the majority of the sources prior to beginning the review, but we used an iterative approach to identify additional sources as they were discovered. We searched and browsed key health sciences library-related journal tables of contents for 2010-2021/2 [31]. We searched all conference proceedings for the Medical Library Association's (MLA) Annual Meetings from 2001-2022 and all available MLA chapter meeting proceedings from 2011-2022. We identified and searched multiple websites and conference proceedings for other relevant organizations. We used Google and Internet Archive's Wayback Machine to search for archived NNLM materials [32]. We reviewed bibliographies of included and relevant journal articles [33], which enabled us to locate the proceedings from the 2006 Conference on Native American Health Information Services in the United States. Finally, we reached out to colleagues who might be able to identify other unpublished materials via mailing lists and personal contacts. Complete details of our grey literature searching, including resource links, are available on our OSF project site [34].

### Screening and Extraction

We screened results from the seven bibliographic databases and E-LIS using a two-stage process in Covidence [35]. We first screened all titles and abstracts and then screened the full text. All results from these eight sources were screened in duplicate by two reviewers in both stages. All non-English language articles were screened by at least one team member able to read the language. Any conflicts were resolved through discussion.

To screen results from other information sources, titles and links for all identified potential materials were added to a shared Google Sheet. Full-text results were screened by one team member and decisions for inclusion or exclusion were verified by an additional team member.

To extract data, we used Google Sheets. One team member extracted data from each included report, and the data was verified by two additional team members. Data elements included bibliographic information and project details (see Table 1). In addition, team members jointly categorized each report by one of the five levels of public participation as described in the International Association for Public Participation (IAP2) Spectrum of Public Participation [36]. The five levels, from least public participation to greatest public participation, are Inform, Consult, Involve, Collaboration, and Empower [36]. Extracted data from report(s) that described the same project(s) were then grouped by project and the IAP2 categorizations were reassessed to reflect the overall project instead of an individual report.

**Table 1:** Categories for Data Extraction

Bibliographic Information	Project Details	Additional Evaluation
Year Title Author(s) Project* DOI or URL	Names of involved organizations Organization types* Geographic location(s) Tribe name(s) if available Other population characteristics Type of intervention/program* Project funding source(s) Length of project Results Was evaluation done? If evaluation was present, how was it done? Type of health issue addressed	IAP2 Categorization**

Table 1. Data extracted from each included report.

* Categorizations were developed as identified and codified into a data dictionary.

** Categorizations were based on definitions by the International Association of Public Participation's (IAP2) Spectrum of Public Participation [36], which was used with permission of the IAP2.

The IAP2 spectrum was “designed to assist with the selection of the level of participation that defines the public's role in any public participation process” [36]. For the purposes of this scoping review, the categorization along the IAP2 spectrum was defined by the participation of the community with which the programs were designed to engage. The participants, therefore, may not be the public, but instead could be medical students, community health workers, or other librarians, so long as the participants were members of, or were ultimately conducting outreach or engagement to, the included populations. To understand the levels of participation reached with identified past engagement and outreach programs, we categorized programs using the IAP2 Spectrum. This categorization helps us determine how much influence the community in focus had on the program and its outcomes. The level of input and involvement community members have on the programs which include them ultimately influences the effectiveness and sustainability of these programs, as demonstrated by initiatives like community-based participatory research [37]. A systematic review of community participation in health services development also found supporting evidence that community involvement positively impacted health outcomes [38]. IAP2 has been used in a similar way by a previous review which assessed child and family engagement in selection of patient reported outcomes for clinical studies [39].

### Data Analysis

To analyze the data, basic descriptive statistics were calculated using Microsoft Excel and Google Sheets. We summarized programs falling within the two highest levels of the IAP2 Spectrum of Public Participation, Collaborate and Empower, to identify key components of community engagement [36]. For these programs, we synthesized any lessons learned as described by the authors of the materials on each program.

## RESULTS

**Figure 1:**
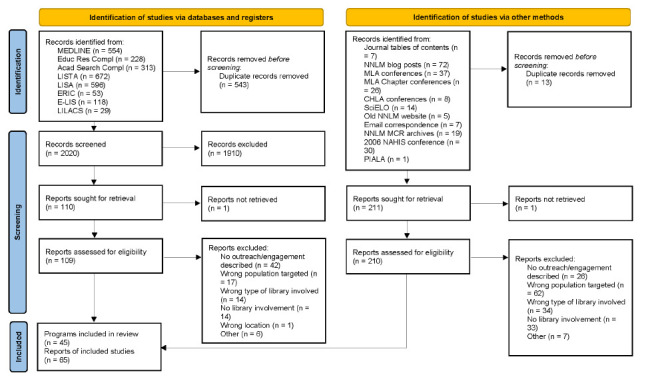
PRISMA 2020 Flow Diagram [17] of Program Identification Process

Database searches resulted in 2,563 results, 543 of which were removed after deduplication, leaving 2,020 for title and abstract screening. 1,910 were excluded based on the determined inclusion/exclusion criteria, whereas 110 were sought for full-text screening. Of the 109 located full-text reports, 94 were excluded upon review. 224 reports were identified through other sources, 13 of which were duplicates; 161 were excluded. 65 total reports were included, representing 45 different programs (see Figure 1). Complete information on included and excluded reports, with reasons for exclusion, is available on the project's OSF site [40].

**Figure 2:**
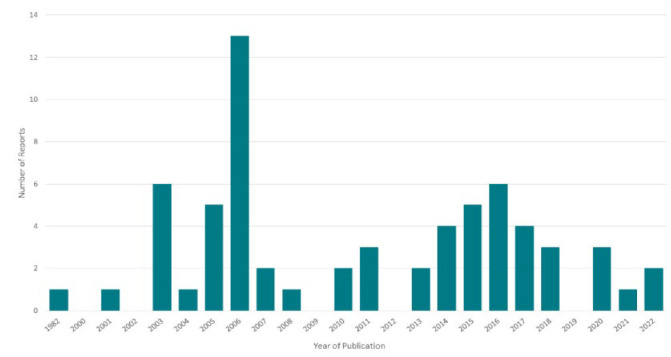
Number of Reports by Year of Publication

Included reports spanned a 40-year time frame, from 1982 to 2022, with the highest number of reports published in 2006 (n=13, 20.0%; see Figure 2). 29 reports (44.6%) were published from 2000-2009; an identical number was published in the following decade (2010-2019). 6 reports (9.2%) have been published since 2020.

Academic health sciences libraries undertook the 45 included programs with 28 different types of organizations. The five most frequent types of organizational partners are included in Table 2. Most libraries had one or more partner for their program (n=39, 86.7%), though six (13.3%) did not have any named organizational partners. The highest number of organization partner types for one program was eighteen (Tribal Connections: IV (2002-2005 Four Corners Region)); the majority of programs had one to three organizational partner types involved (n=28, 62.2%).

**Table 2:** Top 5 Types of Partner

Type of Partner	Number of Partners (n=130) n (%)
Governmental Organization, Federal	19 (14.6)
Governmental Organization, Tribal	11 (8.5)
Library, Federal	10 (7.7)
Non-Governmental Organization/Non-Profit Organization, National or International	9 (6.9)
Academic Department, Same Institution	8 (6.2)

Table 2 shows the top five most commonly partnered with organization types. A total of 130 partners were involved in the 45 programs, including programs that partnered with multiple types of organization.

Twenty-seven types of interventions were used within the 45 programs. Table 3 lists the most common intervention types, those used by five or more programs. Programs used between one and fourteen different intervention types, though most frequently, only one intervention type was used within a single program (n=18, 40.0%). Only two programs (4.4%) used more than 4 unique intervention types (Tribal Connections: IV (2002-2005 Four Corners Region), 14 interventions; South Dakota Native American Health Information Partnership (NAHIP), 7 interventions) [12, 41–54]. The most common intervention, training, was used in 27 programs (60.0% of programs; 25.5% of total interventions).

**Table 3:** Most Common Types of Intervention

Type of Intervention	Number of Interventions (n = 106) n (%)
Training	27 (25.5)
Collection Development	7 (6.6)
Exhibit	7 (6.6)
Technology Purchased	6 (5.7)
Website	6 (5.7)
Reference Service	5 (4.7)

Table 3 shows the intervention types used 5 or more times by the included programs. A total of 106 interventions were described by the 45 programs, including programs that used multiple intervention types.

88.9% (n=40) of programs engaged the public at the three lowest levels of public participation on the IAP2 Spectrum of Public Participation (Table 4). *Inform*, the lowest level, defined by the International Association for Public Participation as having the goal to “provide the public with balanced and objective information to assist them in understanding the problem, alternatives, opportunities, and/or solutions” [36], was the level reached by the majority of programs (n=24, 53.3%). Conversely, only one program (Tribal Connections: IV (2002-2005 Four Corners Region) [12, 43–54]) reached the *Empower* level of public participation, where final decision making is placed in the hands of the public, and an additional four (8.9%) were at the *Collaborate* level, defined as a partnership “with the public in each aspect of the decision including the development of alternatives and the identification of the preferred solution” [36]. These included the Information Resource Dissemination for Minority Populations During the COVID-19 Pandemic [55], Arctic Health Website [56–59], NNLM MidContinental Region: Finding and Evaluating Online Health Information [60], and Outreach to North Dakota Native American Communities programs [61–65]. Of the five programs reaching the top two levels of the IAP2 Spectrum, three had six or more organizational partners. Conversely, all six programs with no organizational partners were in the *Inform* level. Table 5 includes data for each included program.

**Table 4:** Programs by IAP2 Spectrum of Public Participation Level

IAP2 Spectrum Level	Programs n (%)
Inform	24 (53.3)
Consult	6 (13.3)
Involve	10 (22.2)
Collaborate	4 (8.9)
Empower	1 (2.2)

**Table 5:** Included Projects with Summary Data

Project Title	Academic Health Sciences Library(ies)	Summary	Other Organizations by Type	Intervention Type	IAP2 Categorization
AHEC Collaboration for Training Healthcare Professionals [66]	University of Wisconsin-Madison Ebling Library for the Health Sciences	Librarians provided train-the-trainer workshops for healthcare professionals affiliated with the Great Lakes Inter-Tribal Council (GLITC) in 2003 and 2005. Workshops included information on the online research process, databases, and other online resources, finding consumer health resources, and evaluating online information.	Health Care Organization/Provider, State; Non-Governmental Organization/Non-Profit Organization, National or International; NonGovernmental Organization/Non-Profit Organization, Native-Affiliated;	Training	Inform
Arctic Health Website [56–59]	University of Alaska-Anchorage Alaska Medical Library	The chairmanship of the Arctic Council being assumed by the United States led to the NIH charging NLM with promoting health in the Arctic. A contract with the University of Alaska-Anchorage Health Sciences Information Service led to the establishment of the Arctic Health Website, for organization and dissemination of health information affecting communities in the Arctic.	Governmental Organization, Federal; Governmental Organization, International; Governmental Organization, State; Health Care Organization/Provider, Local; Health Care Organization/Provider, Tribal; Library, Federal; NonGovernmental Organization/Non-Profit Organization, Native-Affiliated	Steering Committee; Website	Collaborate
Baltimore American Indian Center Pow Wow Exhibit [67]	University of Maryland, Baltimore Health Sciences and Human Services Library	Exhibit at the Baltimore American Indian Center Pow Wow in 2003.		Exhibit	Inform
Big Bang Health Information Literacy: Outreach to Diverse Populations for Future Success [68]	Pacific College of Oriental Medicine	Collaborative health information training program hosted at six San Diego institutions, with an emphasis on underserved communities, including Native Americans.	Community Partner; Health Care Organization/Provider, Local; Library, Public, City or County; Non-Governmental Organization/Non-Profit Organization, Local or State	Exhibit; Needs Assessment; Training; Translation	Inform
Chinle Comprehensive Care Center and Fort Defiance Indian Hospital Trainings [69]	University of Arizona Health Sciences Library; University of New Mexico Health Sciences Library and Informatics Center	Public health nurses at the Chinle Comprehensive Care Center and Fort Defiance Indian Hospital in the Navajo Nation were introduced to PubMed, received basic research skill training, and were shown the University of New Mexico's Native Health Database, as well as other free, reliable health information resources.	Health Care Organization/Provider, Federal or National; Health Care Organization/Provider, Tribal	Training	Inform
Contract with Navajo Area Indian Health Services [70]	University of New Mexico Health Sciences Library and Informatics Center	Component of outreach program included a contract with the Navajo Area Indian Health Service to provide interlibrary loan, document delivery, literature searches, and reference services.	Governmental Organization, Federal; Health Care Organization/Provider, Federal or National	Document Delivery; Interlibrary Loan; Reference Service	Inform
DEI Events [71]	University of the Incarnate Word Medical Library	Hosted and collaborated in multicultural events to promote diversity, equity, and inclusion for students.		Event	Inform
Gates Foundation Health Trainer Project [72]	University of Arizona Health Sciences Library; Other Unnamed Academic Health Sciences Library(ies)	No detail provided	Non-Governmental Organization/Non-Profit Organization, National or International	Funding; Technology Purchased; Training	Involve
Gates Foundation Native American Access to Technology [72, 73]	University of Arizona Health Sciences Library; Other Unnamed Academic Health Sciences Library(ies)	No detail provided	Governmental Organization, State; Library, State; NonGovernmental Organization/Non-Profit Organization, National or International; School, Library Science	Training	Inform
Health Information Outreach to Native Peoples in Northwestern Nevada [74]	University of Nevada, Reno School of Medicine Savitt Medical Library	Online and in person training and outreach on health information resources to Native peoples in Nevada, from grade 8 to seniors.		Training	Inform
Health Sciences Librarianship Assistantship for Library School Students Interested in Serving Latino and Native American Communities [12, 75, 76]	University of Arizona Health Sciences Library	Twenty-five-year-long internships for library school students interested in serving Latino and Native American communities hosted at the University of Arizona Health Sciences Library. Participants developed a positive view of health sciences librarianship through experiential learning. Many became health sciences librarians.	Governmental Organization, Federal; Health Care Organization/Provider, State; Library, Federal; School, Library Science	Evaluation; Internship; Training	Consult
Indigenous Health Collection [77]	University of Manitoba Neil John Maclean Health Sciences Library	The Indigenous Health Collection features over 5,000 items related to the health of First Nations, Metis, and Inuit peoples. Originally established via donation, liaising with indigenous partners has been used to gather suggestions for additional materials to acquire. Communities and nations have provided further donations as well. The collection has been utilized for curriculum development and serves as an important resource for students at faculty at the Max Rady College of Medicine.		Collection Development	Inform
Information Resource Dissemination for Minority Populations During the COVID-19 Pandemic [55]	University of Arizona Health Sciences Library	This information literacy project trained second-year medical students on finding and evaluating reliable health information resources. The students then taught online workshops for community health workers from Latinx and Native American communities. This project sought to expand upon preexisting Community Engagement Alliance (CEAL) efforts.	Governmental Organization, Federal	Training	Collaborate
Max Rady College of Medicine Indigenous Health Longitudinal Course [77]	University of Manitoba Neil John Maclean Health Sciences Library	Assisted curriculum developers with resources, Indigenous health collections, and LibGuides/bibliographies. Students in 4th year elective component of longitudinal track trained how to find and evaluate information relevant to the patient population beyond MEDLINE, including skills to locate materials created by, or in partnership with, Indigenous people.	School, Medical	Collection Development; Training	Involve
Max Rady College of Medicine Reconciliation Action Plan [77]	University of Manitoba Neil John Maclean Health Sciences Library	Examined Canada's Truth and Reconciliation Commission's calls to action to identify changes that could be made within the institution and library and in roles as leaders, instructors, health care providers, and administrators charged with shaping the next generation of health sciences professionals. Developed areas of action.	School, Medical	Collection Development; Training	Consult
Medical Librarian Research Team Integration [78]	University of North Dakota Health Sciences Library	Embedded into a team providing support for American Indian students who want to go into behavioral health. Provided literature search skills, citation management, and other information skills/knowledge training and guidance.	Academic Department, Native-Affiliated; Academic Department, Same Institution	Reference Service; Training	Inform
Mini-école de la santé [79–82]	Université de Montréal Bibliothèque de la santé	Library partnership with a mini-medical school program enabled the purchase of book collections for First Nations primary and secondary schools in Quebec and Ontario. Books with Indigenous content relating to health and science, and schools were able to select which ones they wanted. Library school students and librarians participated in the mini-medical school program in person.	Academic Department, Same Institution; Health Care Organization/Provider, Local; Non-Governmental Organization/Non-Profit Organization, National or International; School, Elementary; School, High; School, Medical; School, Tribal	Collection Development	Involve
Native American Health Information Services [83]	University of New Mexico Health Sciences Library and Informatics Center	Full-time faculty position for Native American Health Information Services described with first year goals outlined. Initial projects and goals included logic model for evaluation, designing a brochure, and beginning design of a web site. Primary audience for first year was Native American faculty, staff, and students on the University of New Mexico campus.		Database; Evaluation; Website	Inform
Native Cancer Information Resource Center and Learning Exchange (CIRCLE) [84]	Mayo Clinic Libraries	Disseminated copies of curated materials to American Indian/Native American communities, patients, and their providers. Librarians also collaborated to ensure culturally sensitive materials and provide education services.	Academic Department, Same Institution; Health Care Organization/Provider, Local	Document Delivery; Educational Resources; Reference Service	Inform
Native Health Database [44]	University of New Mexico Health Sciences Library and Informatics Center	Database with historical and contemporary Native health/medical information. Includes some information provided partners including the State of New Mexico and the Indian Health Service.	Governmental Organization, Federal; Governmental Organization, State; Health Care Organization/Provider, Federal or National	Database	Inform
Native Investigators [85]	University of Washington Health Sciences Library; University of Colorado Strauss Health Sciences Library	Librarians trained Native Investigators in health literacy.	Academic Department, Native-Affiliated; Academic Department, Same Institution; Health Care Organization/Provider, Local	Training	Consult
Native Voices [86]	UCLA Louise M. Darling Biomedical Library	Hosted Native Voices exhibit and an accompanying panel discussion on traditional and cultural healing practices, environmental health, and contemporary health care issues and policy.		Exhibit; Presentation	Inform
Native Voices [87]	University of New Mexico Health Sciences Library and Informatics Center	Description of the Native Voices exhibit and associated events. Hosted a day-long summit on New Mexico American Indians' concepts of health and wellness with attendees from Navajo, Apache, and Pueblo communities. Exhibit traveled the state. Developed an activity resource kit to provide models of community health and wellness projects that could be adapted by other communities.	Governmental Organization, Tribal; School, Higher Education	Activity Kits; Conference; Exhibit	Involve
Native Voices [88]	University of Washington Health Sciences Library	Brief description of Native Voices traveling exhibit and related events, including an opening blessing and a talk by a member of the Seattle Indian Health Board	Governmental Organization, Federal; Health Care Organization/Provider, Tribal; Library, Federal	Exhibit; Presentation	Inform
Native Voices Exhibit Presentation at Dine College [89]	University of Arizona Health Sciences Library; University of New Mexico Health Sciences Library and Informatics Center	Presentation on Native Voices exhibit, and iPad with Native Voices exhibit information given to Dine College Library. Discussed health sciences librarianship as career.	Library, Tribal College; School, Higher Education; School, Tribal	Presentation	Inform
NLM: Native Internship Pilot Project “Sacred Root” [12, 52]	University of North Dakota Health Sciences Library	Regional medical libraries hosted interns as part of a larger NLM internship program. Libraries supported interns' projects.	Non-Governmental Organization/Non-Profit Organization, National or International	Internship; Technology Purchased; Training	Involve
NLM: Native Voices [9092]	University of New Mexico Health Sciences Library and Informatics Center; University of Washington Health Sciences Library; University of Arizona Health Sciences Library; University of Hawaii at Manoa John A. Burns School of Medicine Health Sciences Library; Other Unnamed Academic Health Sciences Library(ies)	Native Voices exhibit traveled the country, stopping at many academic health sciences libraries. Some libraries planned additional events to go with the exhibit. Host libraries were encouraged to partner with local Indigenous communities when planning events and opening ceremonies.	Governmental Organization, Federal; Library, Federal; Library, Public, City or County; Library, Tribal; NonGovernmental Organization/Non-Profit Organization, National or International;	Exhibit; Site Visit	Involve
NNLM MidContinental Region: Finding and Evaluating Online Health Information [60]	Creighton University Health Sciences Library; University of Kansas Archie Dykes Library	Using a train-the-trainer model, taught basic information about computers and the Internet as well as finding and evaluating online health information to an American Indian tribe. Created materials suitable for later use and adaptation.	Community Partner, Native-Affiliated; Governmental Organization, Federal; NonGovernmental Organization/Non-Profit Organization, National or International	Training	Collaborate
Outreach to North Dakota Native American Communities [12, 61–65]	University of North Dakota Health Sciences Library	Multipronged outreach project including hands-on trainings, demonstrations, and informational sessions on finding quality health information aimed at Native American health care providers and tribal college librarians; purchase of computers and databases for tribal college libraries; development of a web site; and listening circles.	Governmental Organization, Federal; Governmental Organization, Tribal; Health Care Organization/Provider, Federal or National; Library, Tribal College; School, Higher Education; School, Tribal	Database; Technology Purchased; Training; Website	Collaborate
Partnership with Arizona Telemedicine Program [93, 94]	University of Arizona Health Sciences Library	The collaboration between the University of Arizona Health Sciences Library and the Arizona Telemedicine Program allowed the library to disseminate health information to practitioners and consumers in rural areas across Arizona, including tribal Native American communities. The library provided: a health information web portal, ATP site membership in the Arizona Health Information Network (AZHIN), design of the ATP website, facilitation of training to rural site, consumer health information development, and organization of a statewide conference.	Academic Department, Same Institution; Governmental Organization, Tribal; Health Care Organization/Provider, State; Non-Governmental Organization/Non-Profit Organization, Local or State	Conference; Relationship Building; Training; Website	Inform
Poarch Band of Creek Indians Tribal Community Speech Pathology and Audiology Workshop [95]	University of Southern Alabama Charles M. Baugh Biomedical Library	Multidisciplinary collaboration to provide vision and hearing screenings and information about hearing and vision loss.	Academic Department, Same Institution; Library, Higher Education; Non-Governmental Organization/Non-Profit Organization, Local or State	Health Screening; Training	Inform
Poarch Band of Creek Indians Tribal Community Stroke Workshop [96]	University of Southern Alabama Charles M. Baugh Biomedical Library	Multidisciplinary collaboration to provide a workshop on stroke. Workshop included information and hands-on practice searching and using MedlinePlus.	Health Care Organization/Provider, State; Library, Higher Education	Training	Inform
Public Health Workshops [72, 93]	University of Arizona Health Sciences Library	Public health workshops were presented to public health nurses at the Gila River Indian community and in Tuba City on the Navajo Nation.	Governmental Organization, Tribal; Non-Governmental Organization/Non-Profit Organization, Native-Affiliated	Training	Inform
Salud y Bienestar: Entrenamiento Para Promotors / Health and Wellness: Training for Promoters (Community Health Workers) [97, 98]	Burrell College of Osteopathic Medicine Health Sciences Library	Trained community health workers (Promotores) in finding health information, evaluating health information websites, and finding statistics. Sessions took place along the border region, including with the Community Health Representative Program on the Mescalero Apache Reservation. Suicide and HIV/AIDS were topics selected by the communities.	Governmental Organization, Federal; Governmental Organization, State; Governmental Organization, Tribal; Health Care Organization/Provider, State; School, Higher Education	Training	Inform
South Dakota Biomedical Research Infrastructure Network [99]	Roseman University of Health Sciences, Health Sciences, Library; Unnamed South Dakota Health Sciences Libraries	Community of practice of librarians established as a component of the South Dakota Biomedical Research Infrastructure Network. The grant allows for the purchase of scholarly research resources for the entire network. Lead institution librarians train tribal college librarians to access and use biomedical information resources.	Governmental Organization, Federal; School, Higher Education; School, Tribal	Educational Resources; Reference Service; Training	Involve
South Dakota Native American Health Information Partnership (NAHIP) [12, 41, 42]	University of South Dakota School of Medicine, Lommen Health Sciences Library	Connected with four tribal college libraries and community health leaders in five remaining South Dakota reservations. Included training on health information resources and Loansome Doc, document delivery, and reference services with monthly calls to librarians/community health leaders to maintain relationships.	Governmental Organization, Federal; Governmental Organization, Tribal; Library, Tribal College; School, Higher Education; School, Tribal	Collection Development; Document Delivery; Reference Service; Technology Purchased; Training; Translation; Website	Involve
Talking Leaves Collection [100]	Oklahoma State University College of Osteopathic Medicine Medical Library	A collection of curated print books pertaining to the Cherokee Nation and all other Indian tribes in the U.S. (Native American, Alaskan Native, and Native Hawaiian) with a special focus on Native health and medicine. All native and tribal topics are included in the collection.	Governmental Organization, Tribal	Collection Development	Involve
Traditional and Indigenous Healing Collection [101]	University of Alberta John W. Scott Health Sciences Library	The J.W. Scott Health Sciences Library and the University of Alberta's Centre for Cross-Cultural Health and Healing worked together to establish a library collection focused on Traditional and Indigenous Healing, which was formed from materials already owned by the CCCHH and additional materials purchased with funds donated by a CCCHH board member.	Academic Department, Same Institution	Collection Development	Inform
Tribal Connections: I (1998–2000, Pacific Northwest) [12, 52, 73, 102, 103]	University of Washington Health Sciences Library	Worked with an advisory group to identify infrastructure needs in tribal communities in the Pacific Northwest. Technical infrastructure improvements made in 15 of 16 identified sites and training provided on internet basics.	Governmental Organization, Federal; Governmental Organization, Tribal; Library, Federal	Needs Assessment; Steering Committee; Technical Infrastructure; Training	Consult
Tribal Connections: II (2000–2001, Pacific Southwest) [12, 72, 73]	University of Washington Health Sciences Library; University of Arizona Health Sciences Library	Four sites in the Pacific Southwest (two in New Mexico and one each in Arizona and Nevada/ Idaho) participated. Included a needs assessment and improvement of internet connectivity.	Governmental Organization, Federal; Governmental Organization, Tribal; Library, Federal	Technical Infrastructure; Training	Consult
Tribal Connections: III (2001–2003, Pacific Northwest) [12, 52]	University of Washington Health Sciences Library	Project to increase internet connectivity in Native American communities in the Pacific Northwest. Success in 2 of 3 communities.	Governmental Organization, Federal; Governmental Organization, Tribal; Library, Federal	Evaluation; Technical Support	Involve
Tribal Connections: IV (2002–2005 Four Corners Region) [12, 43–54]	University of Colorado Denison Memorial Library; University of New Mexico Health Sciences Library and Informatics Center; University of Utah Spencer S. Eccles Health Sciences Library; University of Arizona Health Sciences Library; UCLA Louise M. Darling Biomedical Library; *The Texas Medical Center Library	Decade-long collaborative effort between Four Corners libraries, their respective Regional Medical Libraries, and multiple external organizations. Used 18 intervention types throughout the efforts. Began with a needs assessment of health care professionals serving Native American communities, contacts database, and web site. Over time, created trust and sustained relationships within communities.	Academic Department, Native-Affiliated; Academic Department, Same Institution; Governmental Organization, Federal; Governmental Organization, State; Governmental Organization, Tribal; Health Care Organization/Provider, Federal or National; Health Care Organization/Provider, Local; Health Care Organization/Provider, Tribal; Library, Federal; Library, Public, City or County; Library, Tribal; Library, Tribal College; Non-Governmental Organization/Non-Profit Organization, Local or State; Non-Governmental Organization/Non-Profit Organization, National or International; NonGovernmental Organization/Non-Profit Organization, Native-Affiliated; Other Partner; School, Higher Education; School, Tribal	Conference; Database; Educational Resources; Event; Exhibit; Library Position Established; Needs Assessment; Presentation; Relationship Building; Site Visit; Technology Purchased; Training; Translation; Website	Empower
Tribal Connections: Special Project, “On Eagles' Wings/Piscataway” [12]	University of Maryland, Baltimore Health Sciences and Human Services Library	Five training sessions conducted by library as an adjunct to a federal project to increase internet infrastructure for Piscataway community in Maryland.	Governmental Organization, Federal; Library, Federal	Funding; Technology Purchased; Training	Consult
Tribal Connections: Steering Committee [73]	University of Arizona Health Sciences Library; University of Washington Health Sciences Library; UCLA Louise M. Darling Biomedical Library; University of Utah Spencer S. Eccles Health Sciences Library; University of New Mexico Health Sciences Library and Informatics Center; *University of Colorado Denison Memorial Library	Steering committee established by resource libraries with Tribal Connections and/or Gates Foundation-funded projects in tribal communities. Met monthly, created a web site to share information, collaborated on funding proposals, and worked to create an asset inventory that would serve as the basis of an effective practices database.	Governmental Organization, Federal; Library, Federal	Evaluation; Steering Committee	Inform
Tribal Librarians' Health Information Conference [93, 104]	University of Arizona Health Sciences Library	Hosted a two-day conference for librarians serving tribal communities on using the Internet effectively to find reliable health information. Used pretest/posttest methodology to gauge use of certain resources to find health information in combination with an 18 month post-conference survey.	Governmental Organization, Federal; Library, State; NonGovernmental Organization/Non-Profit Organization, National or International	Conference; Training	Inform

Table 4 lists the number of programs corresponding with each level of the IAP2 Spectrum of Public Participation [36]. 45 total programs were included.

### Programs and Lessons

Five programs reached either the “empower” or “collaborate” level of public participation. These five programs are summarized in more detail below, along with lessons learned described within the reports.

#### Empower: Tribal Connections Four Corners

The only project identified that reached the “empower” level of public participation was Tribal Connections Four Corners, or TC4C [12, 43–54]. TC4C (also called Tribal Connections IV in some resources) resulted directly from Tribal Connections I-III, NLM funded Native American focused outreach initiatives [12, 52, 72, 73, 102, 103]. TC4C focused on the Four Corners region (New Mexico, Colorado, Utah, and Arizona) in a cross-regional, collaborative project with four main objectives. TC4C involved three Regional Medical Libraries (RMLs) and four academic health sciences libraries, one per state. TC4C's objectives included conducting needs assessments, creating a contacts database, developing a Go Local website for the region, and identifying lessons learned [48]. Each of these objectives had its own working group, and a project administration working group was also established. Additional outreach efforts, such as presenting at the Navajo Nation Fair, were also a component. Monthly or bimonthly teleconferencing was held including all project members, and individual workgroups also met independently by teleconference as needed [48].

In addition to directly funding TC4C's own efforts, additional projects developed by community health organizations and health care professionals were also funded, all of which included partnerships with one or more of the TC4C librarians. For example, the Four Corners Public Library Health Sciences Information Infrastructure project was a partnership between TC4C and five public libraries providing programming focused on health information needs of Native American communities [46]. Public librarian participants received training from the TC4C health sciences librarians and learned how to find and evaluate online health information. This project went beyond informational training by next inviting the librarians to conduct their own health-related projects within their communities. The training therefore was used to empower the librarians by directly giving them control over projects for their own communities. Other funded projects all contained elements which empowered their communities while also providing infrastructure, training, and other interventional components [50, 53, 54].

The TC4C team identified many lessons learned from all aspects of their program. For example, one of the project's objectives, to develop a contact database, was completed, but found little use by participants [48]. TC4C collaborators shared multiple lessons specific to their needs assessment, which required significant relationship building, community knowledge, clear communication, strong leadership, and patience to accomplish. They also noted the importance of having individuals who were Native American or who had experience working with Native American communities on the team for the overall project [48]. The primary librarian for TC4C (funded in part by all the libraries) noted five main lessons: 1) working with tribal communities requires time (in years, not months), patience to build trust, and sustainability; 2) the priorities of tribal communities are not the priorities of academic institutions; 3) there is a wide range of infrastructure and capacities in tribal communities; 4) partner with organizations with similar goals and missions to optimize results; and 5) listen [44, 48].

#### Collaborate: Information Resource Dissemination for Minority Populations During the COVID-19 Pandemic

The University of Arizona Health Sciences Library received NNLM Pacific Southwest Region funding to undertake a multi-tiered information literacy instruction and training initiative [55]. Second-year medical students received training on the evaluation of health information resources from a team of librarians. Recruits for the training sessions were members of the Latinx and Native American communities that they served. These students then conducted workshops for community health workers who also serve Latinx and Native American communities. This collaborative approach therefore included partnership with the target communities at multiple levels, using a train-the-trainer approach that incorporated medical student expertise and community health workers' knowledge. The project used reliable, culturally appropriate information from government sources including NLM, NIH, and the CDC. Sessions were conducted using the Tucson Mexican Consulate's Facebook Live channel. Radio broadcasts and infographic handouts were also developed for Native American communities. The project team worked to identify health network contacts that built upon the Community Engagement Alliance (CEAL) efforts, an NIH initiative [55].

#### Collaborate: Arctic Health Website

A website hosted by the Alaska Medical Library, University of Alaska-Anchorage and originally funded by NLM provides Indigenous health information specifically for Alaska Natives and the circumpolar North [56–59]. The Arctic Health Website formed a collaborative Users Council in its first year which involved participants from NIH, the Alaska Native Science Commission, Alaska's largest hospital, the Alaskan Epidemiology department, Dinlishla, the Alaska Native Tribal Health Consortium, the local CDC office, University of Alaska-Anchorage, and AFHCAN (Alaska Federal Health Care Access Network) [56]. The website was formed with the goal to improve quality health information for Alaska Natives, and through its development and the collaboration of the Users Council, grew to contain publication and research databases, grey literature, photographs and videos, increased consumer health materials, and information on climate change and traditional healing [57, 59].

#### Collaborate: NNLM MidContinental Region: Finding and Evaluating Online Health Information

A program focusing on Community Technology Centers' member organizations and members of an unnamed rural Native American tribe sought to provide training on using computers and the Internet, as well as training on finding and evaluating health information online [60]. The program used a train-the-trainer approach to give community gatekeepers the opportunity to educate their own community members. The program was also deemed suitable for self-study. The training modules were available online and by CD-ROM [60].

#### Collaborate: Outreach to North Dakota Native American Communities

Led by the Harley E. French Library of the Health Sciences at University of North Dakota (UND) and funded through the NNLM Greater Midwest Region, this program encompassed multiple stages and intervention types [61–65]. Initially, the program focused on North Dakota's five tribal college libraries [61]. Health sciences librarians trained their tribal college librarian colleagues, purchased computers for those libraries, funded a subscription for a full-text health database, and established the “Linking Native Americans to Health Information” website [65]. Additional funding enabled training on access to online health information to be provided to the Fort Berthold Reservation Community Health Representatives. This training was also a pilot project to prepare for the second stage of the program [65], which encouraged participation of and collaboration with tribal college librarians throughout the four North Dakota reservations. The program had three main objectives: training health care providers; reinforcing, strengthening, and maintaining relationships with tribal college librarians; and expanding the website “Linking Native Americans to Health Information” [61]. The program included outreach to health care providers at Indian Health Service facilities, health care providers on reservations, and community health representatives or tribal health care personnel working directly with people living on the reservations [63].

The team at UND identified multiple lessons learned throughout the duration of their program. They noted that the “collaboration was accomplished through sensitivity to cultural issues and consistent personal contact” [64]. Similar to the TC4C project lessons, they emphasized listening, flexibility, and seeking advice from Native Americans. At the time, they noted that health care providers and tribal college librarians appreciate training to find quality health information, especially on websites focusing on Native American health problems. Scheduling challenges and tribal politics were also noted as potential barriers [63, 65].

## DISCUSSION

Outreach and engagement programs require an approach to collaboration, labor, and planning which is influenced strongly by preexisting interpersonal and intercommunal relationships and tensions. Approaching outreach and engagement with Native American, Alaska Natives, First Nations, and Indigenous peoples requires recognition of the systemic racism which has perpetuated gaps in health information access and health literacy for these populations. The 45 programs we found through this scoping review are evidence of librarians' efforts to bridge those gaps, often through training on health information resources.

While these programs intended to provide equitable access to health information, the majority of them (30 of the 45 found) did not go beyond the two lowest levels of the IAP Spectrum of Public Participation: inform and consult [36]. According to Arnstein's ladder of citizen participation, informing and consultation are equal to tokenism [105]. Tokenism is defined by citizens (research participants) having opportunities to hear and to be heard. However, under the conditions of informing and consulting the participants ultimately “lack the power to ensure that their opinions will be heeded by the powerful” [105]. Libraries likely struggle to reach further levels of public participation due to long-standing issues with sustainability, which are exacerbated through time-constraints, difficulties with partnerships, need for staffing, and difficulties with securing long-term funding. It is also notable that Wood et al. (2005) stated that computers, internet access, and infrastructure were sustainable while training programs identified in the literature had mixed results [12].

Ultimately, outreach and engagement should strive for the highest level of participation, empowerment. This ensures that the results of a project are as effective, lasting, appropriate, and meaningful for the community as possible. The one project we found that reached the empowerment level of participation was the TC4C or Tribal Connections Four Corners project [12, 43–54]. TC4C demonstrates the importance of establishing infrastructure, and the potential for other types of intervention when some of the identified limitations are overcome.

TC4C empowered through the purchasing of laptops and establishment of a focused outreach role [44, 48]. Needs assessment was one of the primary objectives, meaning understanding the needs of the population was recognized as a necessity from the formation of the program [49]. The expertise of the health sciences librarians was utilized through training, but the knowledge of community partners was appreciated, with program leadership being handed over to community practitioners and other partners [46]. Large and well-funded, with multiple partners and a variety of approaches, TC4C was able to create culturally appropriate resources and provide lasting resources, as well as fund limited outreach projects to reach specific community needs [50, 53, 54].

Partnerships were critical to the success of TC4C. For example, Perry (2006) stated that “health information outreach targeting Native Americans in Colorado is conducted exclusively in the context of participation, with the other “Four Corners” academic health sciences libraries, in the Tribal Connections Four Corners (TC4C) initiative” [51]. Not only was TC4C empowering to the populations it served, it also provided the means for a partnering institution to participate in outreach to their communities when it may not have been possible otherwise. Establishing partnerships does not happen automatically, and requires effort and coordination which also requires time, money, and staff. TC4C itself recorded the difficulties with collaboration they faced, including communication problems, incompatible priorities, and the need for respect of cultural differences [48, 49].

A smaller number of programs with populations outside of the United States were identified. Few Canadian populations were represented [77, 79–82, 101], and no results from Mexican institutions were found, despite the effort made to include Indigenous populations for Canada and Mexico specifically. Exploration of cross-border, multinational collaboration would help to fill these gaps in representation, such as the collaboration demonstrated in the Arctic Health Website project [56–59]. Although this type of collaboration would be challenging, reaching across the boundaries established through colonization is necessary for understanding and working with communities which have been displaced and separated by them.

We can also look to the National Institute on Minority Health and Health Disparities (NIMHD) to further support an approach to engagement which brings the community in at the beginning to specifically work to address what the community wants implemented. The NIMHD's Community-Based Participatory Research program aims to “enhance community capacity by supporting equal community participation in research for which they will directly benefit” [37]. They also directly state a goal to create sustainable programs and to design culturally tailored interventions. These goals should be in line with those of academic health sciences libraries seeking to engage with underserved populations. The CBPR program “begins with involvement of and a research topic of importance to the community” in order to promote health equity. Libraries can begin their outreach and engagement programs with needs assessments, involve the community, and improve the level of participation they reach with this approach [37].

Reflecting on the shift in the information sciences needs of Native American, Alaska Native, First Nations and Indigenous peoples, a focus on data sovereignty could help libraries better align their outreach and engagement to meet the needs of these populations and to move past the lower levels of participation. Transitioning an emphasis on data sovereignty could be a means to “advance research with and for—and not on—Indigenous communities” [106]. It would represent a move to respect for the needs of these populations and a shift of power away from the institutions in which libraries are situated, and into the hands of community members. Though we did not find evidence of academic health sciences libraries engaging communities around data sovereignty topics in this review, this has been a topic undertaken by many academic libraries [107, 108]. In addition, we know that the University of New Mexico's Native Health Database is transitioning to a model providing communities control over their own data [44, 109].

This shift in power and focus to Native American, Alaska Native, First Nations, and Indigenous ownership of research, resources, and outcomes would require a shift in practice. Looking to TC4C as an example, this shift may look like prioritization of needs assessment to better understand the community with which the library seeks to engage. This shift would also mean bringing community stakeholders in from the beginning of engagement to establish relationships, utilize expertise, and prepare a project which can be sustained by community members. This requires compensating community stakeholders for their time and labor.

Compensation for community stakeholders is one small issue in an overall bleak picture of funding identified by this review. In order to reach the furthest levels of participation and create sustainable programs, intention and planning must be backed up by funding at appropriate levels and for a sufficient period of time. It is distinctly noticeable how the National Library of Medicine's episodic funding opportunities and investments have shaped academic health sciences libraries' engagement with Native American and Alaska Native communities. The spike in engagement and outreach we observed from 2003-2006 coincided with NLM's Native American Outreach gaining programmatic status in 2004 [12]. Furthermore, NLM support made Tribal Connections [12, 43–54, 72, 73, 102, 103] and the Native Voices exhibits [86–88, 90–92], the two most widespread programs identified, possible. NLM funding distributed via NNLM regions was crucial to many local programs. In fact, the Regional Medical Libraries of the NNLM oversaw and participated in a large portion of the programs.

Governmental focus has driven engagement and outreach efforts to Native American, Alaska Native, First Nations, and Indigenous communities. Along with the work sponsored directly or indirectly by the National Library of Medicine in the United States, Canada's Truth and Reconciliation Commission work led directly to multiple programs in Canadian academic health sciences libraries. Such efforts included work to build and improve collections related to Indigenous health [77, 101], provide resources for new or expanded courses focusing on Indigenous health [77], and publish about existing programs that demonstrate library commitment to the Truth and Reconciliation Commission's calls to action [80]. The Truth and Reconciliation Commission represents a unique governmental mandate that meaningful research partnerships and programs would work to establish new relationships centered in respect and mutual recognition [110].

Historically, Native American, Alaska Native, First Nations, and Indigenous populations have been taken advantage of through research. As the American Indian Studies scholar, Vine Deloria Jr., described, anthropologists would arrive on reservations, make observations, and author books [111]. These books would then be used to train future anthropologists who would engage in research on reservations, as he states, to verify that the summaries that were written in the books were correct [111]. As recently as 2010, Arizona State University (ASU) settled a lawsuit with the Havasupai Tribe over misuse of genetic samples [112]. As Garrison (2021) found, “The events surrounding ASU's research on the Havasupai distilled existing distrust of medical researchers and discouraged tribe members from participating in further research, even that which might benefit the tribe” [112]. The ethical issues of research and the importance of community engagement that gives a voice to the historically underrepresented are important considerations that must be acknowledged.

As found in this review, librarians and information specialists lack the funding mechanisms and time to create sustainable programs that empower Indigenous populations and may be reminiscent of the anthropologists Deloria Jr. comments upon. Historically, researchers have perpetuated harm through using Indigenous populations to benefit their own research trajectories, not including Indigenous peoples past the consult phase of research, and not delivering on promises made. Librarians with one-time funding may perpetuate harm to Indigenous populations in similar ways, neglecting the importance of continuity, relationship-building, and Indigenous decision-making. One way in which researchers and librarians can look towards creating truly sustainable programs is to partner with other organizations who may be already engaged in or interested in the intended program [113]. Researchers and librarians who wish to improve health disparities through engagement with Indigenous populations should note that sustainability is possible only through continuous tribal engagement. Relying on sporadic small grants from governmental agencies or other funding sources cannot enable the sustained engagement required; academic health sciences libraries interested in this type of engagement should consider prioritizing it in their budgets.

## LIMITATIONS

We recognize that much outreach and engagement work may go unpublished, and thus, this scoping review is highly unlikely to have located all programs that would have fit our criteria. Community engagement and outreach requires a lot of that work that occurs in the background or through the development of a relationship over time. Many times, this means that scholarly articles may simply not be a priority. Indeed, we identified 50 of the 65 articles for inclusion through sources other than databases. With the majority of research literature representing grey literature, there is an overall lack of articles published in academic or peer-reviewed journals.

In addition, the search strategy we used, even though it was an attempt to be as comprehensive as possible, is likely to have missed a lot of material. We know, for example, that the search strategy missed alternate spellings of Ojibwe (Ojibwa, Ojibway), which was brought to our attention by a librarian looking at the published search strategy (E. Kinzel email to A. Cruise, 2022-02-17). She suggested instead truncating Ojibw* to retrieve maximum results, which we did not go back to do. We are certain to have missed many other ways that Native American, Alaska Native, First Nations, and Indigenous communities describe themselves, particularly in the Spanish language information resources. Though we strove to locate and include terms relating to Indigenous communities of Mexico, for example, we were unable to search every information source comprehensively with all of the terms we identified for the main database searches.

Secondly, we acknowledge that the designations of IAP2 Spectrum of Public Participation categorizations were based solely on what we could ascertain from what we read [36]. We often had very limited details (e.g., an abstract, or an unannotated PowerPoint presentation or poster) as our sole piece of information on a program. Most identified reports did not contain full program evaluations, and some in fact were only partial abstracts without results and conclusions. Therefore, how we interpreted those reports is not necessarily a complete or fair representation of what actually happened in the programs. It is perhaps not a surprise that TC4C, the only program we considered in the “Empower” category, had the most reports, and the most complete reports, of any program. We also acknowledge that the majority of identified programs were funded or directed with the lower level performance expectations. IAP2 does point the way for more effective outreach participation levels in the future.

For a more complete picture of outreach involvement and effective partnerships between health science libraries and Native American, Alaska Native, First Nations, and Indigenous communities, conducting first person interviews, focus groups, and/or surveys would bring to light additional details and themes. In addition, there may be examples of successful partnerships between different library groups and other underserved or marginalized populations that a broader review of the literature may uncover.

## CONCLUSION

Academic health sciences libraries were and continue to be involved in community engagement and outreach with Native American, Alaska Native, First Nations, and Indigenous communities, but these efforts require time, long-term commitment, sustained funding. Intentional effort, planning, and attention to culture and history are also necessary to involve communities in ways which will bring about the most effective and meaningful relationships.

Many programs are short-term and conducted at the lowest levels of public participation, but the ephemeral nature of many of the reports we located may have influenced our perceptions and categorizations. We hope with this scoping review to encourage librarians participating in this area to share their work to help bolster interest and activity, and to improve engagement and outreach practices in academic health sciences libraries. Through these efforts, mutual, sustainable engagement and outreach programs can emerge which will empower communities and promote health equity.

## Data Availability

All data from this scoping review is available on Open Science Framework [40].
